# Pre‐hospital management protocols and perceived difficulty in diagnosing acute heart failure

**DOI:** 10.1002/ehf2.12524

**Published:** 2019-11-08

**Authors:** Pia Harjola, Òscar Miró, Francisco J. Martín‐Sánchez, Xavier Escalada, Yonathan Freund, Andrea Penaloza, Michael Christ, David C. Cone, Said Laribi, Markku Kuisma, Tuukka Tarvasmäki, Veli‐Pekka Harjola, on behalf of the EMS‐AHF Study Group

**Affiliations:** ^1^ Emergency Medicine, University of Helsinki, Department of Emergency Medicine and Services Helsinki University Hospital Helsinki Finland; ^2^ Emergency Department, Hospital Clínic University of Barcelona Villarroel 170 Barcelona 08036 Spain; ^3^ Emergency Department, Hospital Clínico San Carlos Instituto de Investigación Sanitaria Hospital Clínico San Carlos (IdISSC), Facultad de Medicina de Universidad Complutense de Madrid Madrid Spain; ^4^ Sistema d'Emergències Mèdiques Tarragona Spain; ^5^ Emergency Department, Hôpital Pitie‐Salpêtrière Assistance Publique‐Hôpitaux de Paris, INSERM 1166, Sorbonne University Paris France; ^6^ Emergency Department, Cliniques Universitaires St‐Luc Université Catholique de Louvain Brussels Belgium; ^7^ Department of Emergency Medicine Luzerner Kantonsspital Lucerne Switzerland; ^8^ Department of Emergency Medicine Yale University School of Medicine New Haven CT USA; ^9^ Département de Médecine d'Urgence, CHRU de Tours Faculté de Médecine, Université de Tours Centre d'Étude des Pathologies Respiratoires ‐ Inserm U1100 Tours France; ^10^ Cardiology, University of Helsinki, Heart and Lung Center Helsinki University Hospital Helsinki Finland

**Keywords:** Acute heart failure, Pre‐hospital, Emergency care, Dispatching centre, Emergency medical services

## Abstract

**Aim:**

To illustrate the pre‐hospital management arsenals and protocols in different EMS units, and to estimate the perceived difficulty of diagnosing suspected acute heart failure (AHF) compared with other common pre‐hospital conditions.

**Methods and results:**

A multinational survey included 104 emergency medical service (EMS) regions from 18 countries. Diagnostic and therapeutic arsenals related to AHF management were reported for each type of EMS unit. The prevalence and contents of management protocols for common medical conditions treated pre‐hospitally was collected. The perceived difficulty of diagnosing AHF and other medical conditions by emergency medical dispatchers and EMS personnel was interrogated.

Ultrasound devices and point‐of‐care testing were available in advanced life support and helicopter EMS units in fewer than 25% of EMS regions. AHF protocols were present in 80.8% of regions. Protocols for ST‐elevation myocardial infarction, chest pain, and dyspnoea were present in 95.2, 80.8, and 76.0% of EMS regions, respectively. Protocolized diagnostic actions for AHF management included 12‐lead electrocardiogram (92.1% of regions), ultrasound examination (16.0%), and point‐of‐care testings for troponin and BNP (6.0 and 3.5%). Therapeutic actions included supplementary oxygen (93.2%), non‐invasive ventilation (80.7%), intravenous furosemide, opiates, nitroglycerine (69.0, 68.6, and 57.0%), and intubation 71.5%. Diagnosing suspected AHF was considered easy to moderate by EMS personnel and moderate to difficult by emergency medical dispatchers (without significant differences between *de novo* and decompensated heart failure). In both settings, diagnosis of suspected AHF was considered easier than pulmonary embolism and more difficult than ST‐elevation myocardial infarction, asthma, and stroke.

**Conclusions:**

The prevalence of AHF protocols is rather high but the contents seem to vary. Difficulty of diagnosing suspected AHF seems to be moderate compared with other pre‐hospital conditions.

## Introduction

Acute heart failure (AHF) is a common medical condition encountered in the emergency departments (ED) and pre‐hospital settings.^1^, ^2^ From 11–53% of AHF patients arrive to the ED by ambulance.^3^, ^4^, ^5^ While the prognosis of AHF patients' remains poor,^6^, ^7^, ^8^, ^9^ the importance of early phase and pre‐hospital management by emergency medical services (EMS) has been recently underlined.^10^, ^11^, ^12^, ^13^

Traditionally, EMS units are categorized by their resources for diagnosis and care and the level of personnel. Typically, advanced life support (ALS) units have a physician, nurse, or paramedic aboard (depending on the country) with readiness for intravenous (IV) line insertion and IV‐medication administration. Most helicopter EMS (HEMS) units also correspond to this category. In contrast, basic life support (BLS) units are usually resourced and staffed for less critical situations.

The routine AHF management includes 12‐lead electrocardiogram (ECG) recording, vital sign monitoring, option for treatment with supplementary oxygen, non‐invasive ventilation (NIV), and administration of IV diuretics and vasodilators.^11^, ^12^ However, the administration of pre‐hospital medication seems scarce.^3^, ^4^, ^13^, ^14^ Moreover, earlier studies suggest that it might be difficult for EMS personnel to differentiate AHF from other underlying causes of dyspnoea^14^, ^15^, ^16^, ^17^ especially when the diagnosis is based only on patient's medical history and clinical signs and symptoms.^18^, ^19^

Illustrative data on the possibilities of EMS to treat and diagnose AHF in the pre‐hospital setting in accordance with the guidelines are scarce. Bearing in mind these gaps in current knowledge, the present study was designed to investigate the possibilities to diagnose and treat AHF in the pre‐hospital setting. We also assessed the prevalence of specific management protocols for dyspnoeic conditions and the perceived difficulty of diagnosing suspected AHF and other critical conditions encountered in EMS.

## Methods

The EMS‐AHF study was based on a multinational survey. The surveys were sent to persons in charge of an EMS region. These regional EMS leaders were contacted by key national emergency physicians who agreed to participate in the present study. Data were collected between November 2017 and February 2018 from 104 EMS regions in 18 countries. Fifteen of these countries were European (Belgium, Czech Republic, Denmark, Estonia, Finland, France, Germany, Italy, Lithuania, Monaco, Norway, Poland, Slovenia, Spain, and Switzerland). In addition, Canada, Singapore, and the United States were included.

The collected data included information about the different types of EMS units and the diagnostic and therapeutic arsenals on board; BLS, ALS, and HEMS units were assessed separately. The availability of diagnostic tools was classified into three categories: 0 = ‘not on board', 1 = ‘in some ambulances', and 2 = ‘in all ambulances'. Likewise, availability of the treatment options was classified as 0 = ‘not available', 1 = ‘permission needed', and 2 = ‘permanent standing order'. In addition, data on the prevalence of specific pre‐hospital management protocols for AHF, dyspnoea, chest pain, and ST‐elevation myocardial infarction (STEMI) were collected. The data included information about diagnostic and therapeutic actions, which were again classified as 0 = ‘not on board', 1 = ‘permission needed', and 2 = ‘permanent standing order'. For statistical analyses, we combined Categories 1 and 2 and compared with Category 0. Finally, the perceived difficulty of diagnosing suspected AHF (distinguishing between *de novo* AHF and acute‐decompensated heart failure [ADHF]) and other common conditions encountered in EMS (stroke, acute coronary syndrome in general, STEMI, asthma attack, pulmonary embolism, and sepsis) were estimated and graded according to a 5‐grade scale: 1 = ‘very easy', 2 = ‘easy', 3 = ‘moderate', 4 = ‘difficult', and 5 = ‘very difficult'. Separated scores grading difficulty of diagnosing suspected AHF by emergency medical dispatchers (EMD) and by EMS personnel were collected.

The categorical variables are presented as absolute values and percentages and compared by Fisher's exact test. Perceived difficulty is presented as a quantitative variable ranging from 1 (minimal difficulty) to 5 (maximal difficulty), reported as mean and standard deviation, and compared by Student's *t*‐test. Statistical analysis was performed using SPPS Version 25 (IBM Corp., Armonk, NY, USA).

## Results

The survey covered more than 20% of the respective country's population in 11 out of 18 countries (Singapore 100.0%, Monaco 97.5%, Spain 79.0%, Estonia 70.5%, Finland 66.2%, Switzerland 39.5%, Lithuania 29.4%, Norway 29.4%, Belgium 27.4%, Denmark 22.7%, and France 21.1%). The population coverage ranged from 2.2–13.2% in Canada, Czech Republic, Germany, Italy, Poland, Slovenia, and United states.

**Figure 1:**
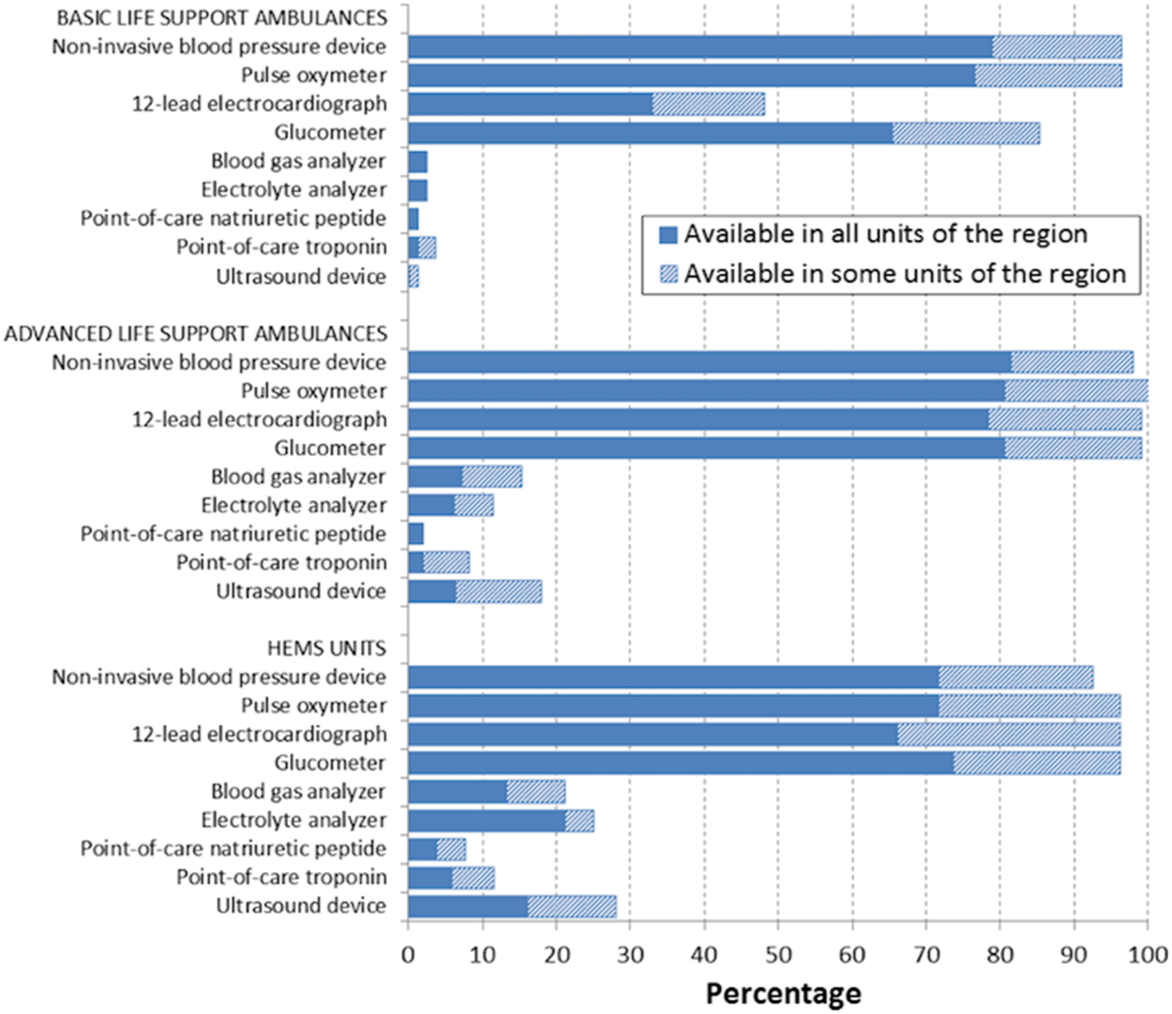
Availability of diagnostic tools in different type of emergency medical service units. HEMS, helicopter emergency medical services.

**Figure 2:**
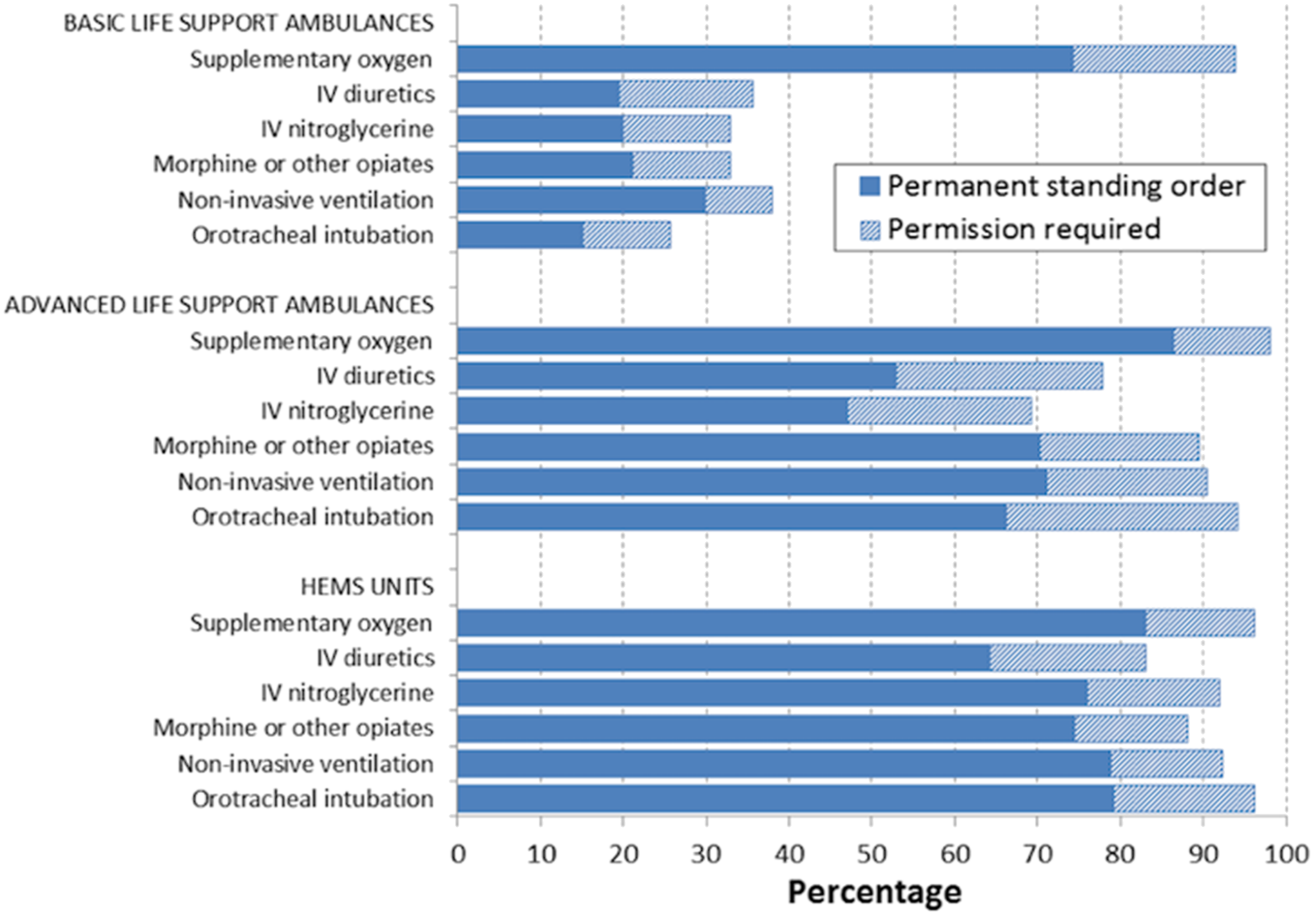
Possibility of administration of common therapeutic treatments for acute heart failure in different emergency medicine service units including those with permanent standing order and those requiring permission. HEMS, helicopter emergency medical services; IV, intravenous.

The capability of EMS personnel to run diagnostic tests (*Figure* *1*) or to provide therapeutic treatments (*Figure* *2*) potentially needed for managing AHF patient largely varied according with the type of EMS unit. The diagnostic and therapeutic arsenals were more commonly available in ALS and HEMS units than in BLS units. However, the availability of point‐of‐care testing (POCT) and ultrasound, even in the ALS and HEMS units, was quite low (less than 25% EMS regions had these). With respect to therapeutic arsenal, apart from supplementary oxygen, AHF treatments were available in roughly one‐third, or less, of BLS units, whereas majority of ALS and HEMS units had these treatment options. Main AHF medications (diuretics and nitroglycerine) were available in approximately half of ALS units as permanent standing order.

A specific protocol for pre‐hospital AHF management was present in 84 regions (80.8%). Prevalence of protocols for chest pain (84 regions, 80.8%, *P* = 1.00) and dyspnoea (79 regions, 76.0%, *P* = 0.50) was similar to AHF protocols. Whereas, the prevalence of STEMI protocols was significantly higher than AHF protocols (99 regions, 95.2%, *P* < 0.001). The contents of the different management protocols are shown in *Table* 1. With respect to diagnostic actions, 12‐lead ECG was included in almost all AHF protocols, whereas POCTs for BNP and troponin were included only in few protocols and ultrasound in 14–16% of protocols. In general, AHF management most commonly—in over 90% of AHF protocols—involved acquisition of 12‐lead ECG, insertion of an IV line, and administration of supplementary oxygen. With respect to therapeutic actions, after supplementary oxygen, NIV was the most common action (present in the AHF protocols of 80.7% EMS regions), followed by intubation (71.5%), IV diuretics (69%), IV opiates (68.6%), and IV nitroglycerine (57.0%). Compared with the non‐specific dyspnoea protocols, AHF protocols more frequently included IV diuretic and nitroglycerine administration; compared with chest pain, IV diuretics and NIV were more frequently recommended. Compared with STEMI protocols, AHF protocols less frequently included ECG and IV opiate administration, and more frequently IV diuretic and NIV use (*Table* 1).

**Table 1:** Protocolized actions contained in the acute heart failure management protocol and in the other three additional pre‐hospital protocols surveyed

	AHF	Dyspnoea	Chest pain	STEMI
	*N* = 84	*N* = 79	*N* = 84	*N* = 99
Diagnostic actions Total % (% permission request needed/% permanent standing order)				
Take a 12‐lead ECG	92.1 (20.5/71.6) ***	84.5 (20.2/64.3)	98.8 (21.8/77.0)	99.0 (19.0/80.0)
Run a POC‐testing for troponin	6.0 (2.4/3.6)	7.2 (3.6/3.6)	8.2 (3.5/4.7)	8.2 (1.0/7.2)
Run a POC‐testing for BNP/NT‐proBNP	3.5 (3.5/0.0)	3.6 (3.6/0.0)	3.5 (3.5/0.0)	2.1 (0.0/2.1)
Do ultrasound	16.0 (5.7/10.3)	15.5 (6.0/9.5)	14.9 (5.7/9.2)	15.0 (4.0/11.0)
Therapeutic actions Total % (% permission request needed/% permanent standing order)				
Insert an IV line	94.3 (21.6/72.7)	90.4 (21.4/69.0)	95.4 (21.8/73.6)	98.0 (20.2/77.8)
Provide supplementary oxygen	93.2 (21.6/71.6)	92.8 (23.8/69.0)	86.2 (17.2/69.0)	87.0 (24.0/63.0)
Provide IV diuretics	69.0(29.9/39.1)* ^,^ ** ^,^ ***	49.4 (24.1/25.3)	32.6 (14.0/18.6)	36.4 (17.2/19.2)
Provide morphine or another opiate	68.6 (22.1/46.5)***	54.9 (19.5/35.4)	80.0 (25.9/54.1)	89.8 (29.6/60.2)
Provide IV nitroglycerine	57.0 (31.4/25.6)*	31.4 (16.9/14.5)	50.6 (18.8/31.8)	60.2 (24.5/35.7)
Provide non‐invasive ventilation	80.7(25.0/55.7)** ^,^ ***	82.2 (28.6/53.6)	45.4 (16.3/29.1)	50.5 (22.8/27.7)
Perform intubation	71.5 (29.5/42.0)	77.3 (33.3/44.0)	64.3 (24.1/40.2)	65.0 (27.0/38.0)

AHF, acute heart failure; BNP, brain natriuretic peptide; ECG, electrocardiogram; IV, intravenous; NT‐proBNP, N terminal pro brain natriuretic peptide; POC, point of care; STEMI, ST‐elevation myocardial infarction.

* *P* < 0.05 in comparison with the dyspnea protocol.

** *P* < 0.05 in comparison with the chest pain protocol.

*** *P* < 0.05 in comparison with the STEMI protocol.

**Figure 3:**
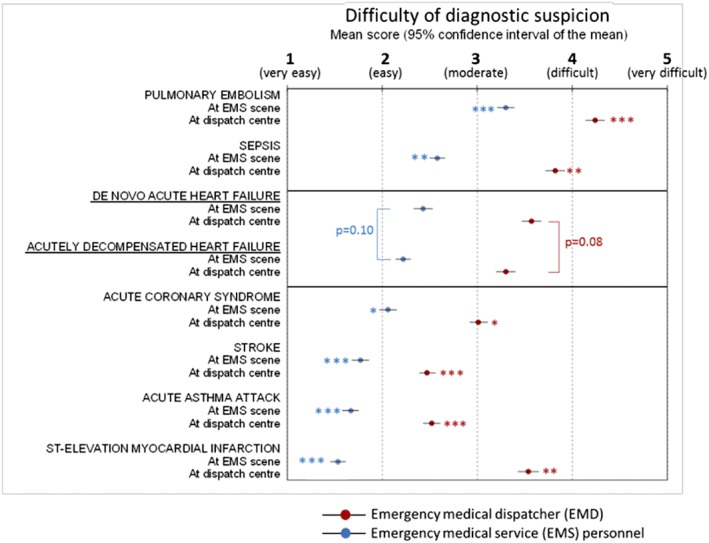
Perceived difficulty of diagnosing suspected acute heart failure (differentiated by *de novo* and decompensated) by emergency centre dispatchers (blue) and by emergency medical service personnel at scene (red). Comparisons were established between pairs in the same setting. ^*^ *P* < 0.05 compared with *de novo* acute heart failure. ^**^ *P* < 0.05 compared with acutely decompensated heart failure.

Surveys about the perceived difficulty of diagnosing suspected AHF by EMS personnel and by EMD were provided by 101 (97.1%) and 96 (92.3%) participants, respectively. They did not report significant differences between diagnostic difficulty of *de novo* AHF and ADHF in either scenario. *De novo* AHF and ADHF were both graded to be easy to moderate to suspect by EMS personnel and moderate to difficult by EMD (*Figure* *3*). For EMD, both types of AHF were significantly more difficult to suspect than stroke and acute asthma and easier than pulmonary embolism. On the other hand, for EMS personnel, AHF (*de novo* and ADHF) was significantly more difficult to suspect than STEMI, stroke, and acute asthma and easier than pulmonary embolism (*Figure* *3*). For all the conditions assessed, suspicion by EMD was always considered to be significantly more difficult than by EMS personnel.

## Discussion

The EMS‐AHF study describes for the first time the pre‐hospital management of AHF from a multinational perspective. Our study provides three main findings. First, though only minority of EMS units carry diagnostic tools that can help in AHF diagnosis, the majority of units have the possibility to provide recommended AHF treatments. Second, AHF management protocols are common in the pre‐hospital setting. Yet, the contents of these protocols vary between EMS regions. Third, diagnosing suspected AHF is perceived to be easy to moderate at scene but moderate to difficult at dispatching centres, with no significant difference reported between ADHF and *de novo* AHF.

The differential diagnosis between AHF and other medical conditions causing dyspnoea is difficult without the use of diagnostic tools^20^ and may lead to misdiagnosis and inappropriate, even harmful, treatment of dyspnoeic patients.^1^ Our survey showed that the prevalence of diagnostic tools is low in the EMS units, even in ALS and HEMS units. Thus, there is room for improvement in the availability of diagnostic tools, such as POCTs for BNP and troponin and ultrasound devices in the EMS units' arsenals.

The availability of therapeutic arsenals varies between different types of EMS units, as could be expected from their roles in the EMS organization. IV diuretics and vasodilators such as nitroglycerine (the mainstay of AHF treatment)^21^ are on board in the majority of ALS and HEMS units, whereas only a minority of BLS units can provide these treatments. Very recent data suggest that early IV diuretic administration may improve mortality in AHF.^9^, ^10^, ^11^, ^12^ Considering these two findings, it is important that EMD provides the most appropriate EMS unit for a patient with suspected AHF. However, the reported proportion of AHF patients calling to dispatching centres varies from 11% in Finland 3 to 53% of cases in Spain,^4^ and less than one‐third of AHF patients are managed by an ALS unit.^3^, ^4^ The SEMICA study (Emergency Medical Response Systems for Patients with Acute Heart Failure) explored factors associated with EMS unit provision by EMD and found out that ALS unit assignment was well related to the severity of AHF.^4^, ^22^

In our survey, most of the EMS regions had a specific management protocol for AHF. The prevalence of AHF protocols was similar to that of chest pain and dyspnoea protocols. Some earlier studies show that delays in AHF management may increase mortality and morbidity.^19^, ^23^ The use of specific management protocols in EMS might reduce the delays in the initiation of pre‐hospital management. The prevalence of AHF management protocols can be considered rather high as the early diagnosis and treatment of AHF have been emphasized somewhat recently.^11^, ^12^, ^13^ As expected, management protocols for STEMI were even more prevalent because of stronger evidence and recommendation in guidelines.^24^ The importance of time to treatment in STEMI is unambiguous, as the condition evolves more abruptly compared with AHF. In contrast, the prognostic significance of time to treatment in AHF still remains controversial.^10^, ^25^ More importantly, AHF manifests in several clinical phenotypes, which vary in their acuity and severity.^26^ Specific causes of AHF like acute coronary syndrome, hypertensive emergency, or arrhythmias justify cause‐specific pre‐hospital management as well. Interestingly, chest pain protocols were no more common than AHF protocols, and dyspnoea protocols were reported only in 76% of EMS regions.

The contents of the management protocols vary between the EMS regions. The prevalence of IV opiates was high in the AHF management protocols, which is somewhat alarming because opiates are not a routine medication for all AHF patients and is recommended to be used with caution.^21^, ^27^ Moreover, IV opiates were included in AHF management protocols more often than IV nitroglycerine, which in contrast is one of the mainstay medications well available in EMS units. A vast majority of AHF protocols included respiratory support, which is an essential part of AHF management, and timely NIV may reduce the need for intubation in pulmonary oedema.^28^

The diagnosis of both *de novo* AHF and ADHF is perceived to be easy to moderate. It was found to be significantly more difficult in the dispatching centres. In comparison, STEMI was considered the easiest pre‐hospital condition to diagnose among all the conditions surveyed, probably because of the rather unequivocal diagnostic ECG criteria based on ST changes and the high availability of 12‐lead ECG. In the case of AHF, the POCT for BNP could play a similar role.

Further studies should be done to evaluate the accuracy of the pre‐hospital AHF diagnosis.

### Limitations

There are some limitations to be acknowledged. First, the national representativeness of the surveys in different countries was variable. Still, we feel that our survey gives an adequate overview of the current status. Second, the diagnostic difficulty in the pre‐hospital setting was based on subjective views of the EMS regional leaders. However, the interview of individual staff members was beyond the scope of the present study.

## Conclusions

The prevalence of AHF protocols is rather high and, AHF‐treatment options, especially respiratory support, are readily available in ALS and HEMS units. The pre‐hospital diagnosis of critical medical conditions is perceived to be significantly more difficult in dispatching centre compared with the EMS at scene. The diagnosis of both ADHF and *de novo* AHF is reported to be easy, even though the prevalence of diagnostic tools in EMS units is scarce. Future studies are warranted to investigate the accuracy of the EMS diagnosis and the cause behind the perceived diagnostic easiness of AHF.

## Conflict of interest

The authors declare no conflict of interest.

## Funding

This work was supported by Department of Emergency Medicine, Helsinki University Hospital, University of Helsinki, Helsingin ja Uudenmaan sairaanhoitopiiri to P.H.
